# Increased Accumulation of Ginsenosides in *Panax ginseng* Sprouts Cultivated with Kelp Fermentates

**DOI:** 10.3390/plants13030463

**Published:** 2024-02-05

**Authors:** Kyung-Wuk Park, Jeong-Ho Kim, Beom-Gyun Jeong, Jun-Ki Park, Ho-Yeol Jang, Yun-Seo Oh, Kyung-Yun Kang

**Affiliations:** R&D Team, Suncheon Research Center for Bio Health Care, Suncheon-si 57962, Republic of Korea; uk988446@sbrc.kr (K.-W.P.); kimjeoho90@gmail.com (J.-H.K.); fusionchef@sbrc.kr (B.-G.J.); nada3663@naver.com (J.-K.P.); yeol2686@naver.com (H.-Y.J.); yun-seo@sbrc.kr (Y.-S.O.)

**Keywords:** ginseng sprout, nutrient solution, hydroponics, kelp fermentate, ginsenoside

## Abstract

Currently, new agri-tech has been developed and adapted for the cultivation of crops using smart farming technologies, e.g., plant factories and hydroponics. Kelp (*Laminaria japonica*), which has a high industrial value, was considered as an alternative to chemicals for its eco-friendly and sustainably wide use in crop cultivation. In this study, a fermented kelp (FK) was developed for use in hydroponics. The FK contained various free and protein-bound amino acid compositions produced by fermenting the kelp with *Saccharomyces cerevisiae*. Supplementing FK as an aeroponic medium when cultivating ginseng sprouts (GSs) elevated the total phenolic and flavonoid contents. Additionally, seven ginsenosides (Rg1, Re, Rb1, Rc, Rg2, Rb2, and Rd) in GSs cultivated with FK in a smart-farm system were identified and quantified by a high-performance liquid chromatography-evaporative light scattering detector/mass spectrometry analysis. Administering FK significantly increased the ginsenosides in the GSs compared to the control group, which was cultivated with tap water. These results indicate the FK administration contributed to the increased accumulation of ginsenosides in the GSs. Overall, this study suggests that FK, which contains abundant nutrients for plant growth, can be used as a novel nutrient solution to enhance the ginsenoside content in GSs during hydroponic cultivation.

## 1. Introduction

*Panax ginseng* Meyer, known as ginseng, is widely used as an herbal medicine in East and Southeast Asia [1]. Many studies have reported on the analysis of chemical constituents in ginseng, such as saponins, flavonoids, amino acids, polysaccharides, and volatile oils [2]. In particular, ginsenosides, which are triterpenoid saponins, are the representative biologically active compounds in ginseng that ameliorate its cardioprotective, neuroprotective, antitumor, anti-inflammatory, and anti-diabetes mellitus characteristics [3,4,5,6,7]. Although there are numerous studies on the biological activities of ginseng, due to its price and long cultivation period, many researchers and related industries have recently been interested in ginseng sprouts (GSs), which could be cultivated quickly while maintaining the quality characteristics [8,9].

The smart farming system provides definitive optimal circumstances for plant growth by controlling cultivation conditions using information and communication technologies [10]. Previous studies have reported on the enhancement of efficiency, effectiveness, and productivity for crops grown with smart farming systems. Tayade et al. [11] reported on the supplementation of silicon as a smart fertilizer for crop cultivation. Cruz et al. [12] suggested connecting and managing the smart farming system using the Internet of Things platform for smart strawberry farming. Modarelli et al. [13] proposed that aquaponics is suitable for leafy crops such as basil (*Ocimum basilicum*) due to the reduced utilization of chemical fertilizers and increased productivity. Kim et al. [14] reported that treating one-year-old ginseng with light-emitting diodes (LEDs) increased the weight of shoots in the cultivation chamber. Several studies have also analyzed the phytochemicals in ginseng cultivated using smart-farm systems. Twelve ginsenosides (Rb1, Rb2, Rc, Rd, Re, etc.) were analyzed in GSs cultivated in an aeroponic system using plasma-treated water as a nitrogen source [15]. The phenolic acid, flavanol, and ginsenoside contents have also been determined for GSs cultivated in soil-substrate and deep-water types of smart farms in a plant factory [1]. However, studies on the development of nutrient solutions for hydroponics to increase bioactive compounds have not been fully explored.

In the last two decades, various efforts have been reported relevant to producing the biologically active compounds contained in ginseng by utilizing microorganisms. The extracellular enzymes from a fungus isolated from fermented soybean brick were determined to produce compound K, which had previously only been produced with biological methods by ginsenosides in a ginseng extract [16]. Similarly, enzymes isolated from recombinant *Lactococcus lactis* efficiently produced ginsenoside Rg3 and increased the conversion yields from ginsenosides to compound K [17]. There is also a report on the bioconversion of ginsenosides with five lactic acid bacteria isolated from kimchi, a traditional Korean fermented food [18]. While most reports have demonstrated enhanced conversion yields from ginsenosides in ginseng extract to compound K by microorganisms, investigations on elevating the bioactive compounds in ginseng by the treatment and administration of a nutrient solution produced by microorganisms are still needed. Hence, in this study, changes in the accumulation of biologically active compounds were investigated to develop a nutrient solution for the cultivation of ginsenoside-enriched GSs.

Many studies have reported that kelp (*Laminaria japonica*) has a high nutritional value and contains abundant phytochemicals [19]. The commercial market for chemical products extracted from macroalgae, especially brown seaweed kelp, has been increasing due to their high value [20]. Due to this increase in the market, seaweed waste that can be utilized as an industrial resource has also been elevated [21]. The chemical composition of kelp is reported to be 13% proteins, 3% fats, 53% carbohydrates, and 31% ash, which indicates an abundant amount of minerals and proteins that could improve plant growth [22,23]. Several studies have reported on the effects of seaweed extract for improving the growth of plants. Kelp waste extracts treated with cellulase, pectinase, and papain were shown to enhance the growth and quality characteristics of *Brassica chinensis* L. [24]. Similarly, a co-treatment with plant-growth-promoting rhizobacteria and kelp extract improved the growth and nutrition content in *Allium cepa* L. [25]. While previous studies have reported on the utilization of kelp extracts as fertilizers and biostimulants, more studies on the development of a nutrient solution utilizing kelp with microorganisms are still required due to its eco-friendly and sustainably wide use in crop cultivation.

In this study, kelp was fermented with *Saccharomyces cerevisiae*, and the physicochemical properties were determined during the fermentation period. Furthermore, changes to the ginsenoside contents in GSs that were administered a nutrient solution of fermented kelp (FK) for hydroponic growth were investigated to evaluate the enhancement of ginsenoside accumulation.

## 2. Results and Discussion

### 2.1. Salt-Reduced Kelp Powder for Fermentation via the Boiling Process

High salinity doses affect the growth of microorganisms in the medium [26]. It has been shown that treating various kinds of dehydrated seaweeds such as *Chondrus crispus*, *Laminaria digitata*, *Saccharina latissimia*, and *Undaria pinnatifida* with a boiling process significantly reduces their Na content [27]. For these reasons, thermal processing was performed to reduce the salt content in the kelp we used. Table 1 shows that the salinity of the raw kelp was 1.97%, which could inhibit the growth of *S. cerevisiae*. After the thermal processing in hot water, the salinity of the kelp was reduced to 0.43%, which would not inhibit the growth of *S. cerevisiae* during fermentation. To develop a nutrient solution for hydroponics, the boiled kelp was dried until its water content was less than 10% and then ground to a powder and stored at −20 °C until use in the experiments.

### 2.2. Changes to Physicochemical Properties during the Production of Fermented Kelp for Hydroponics

**Figure 1:**
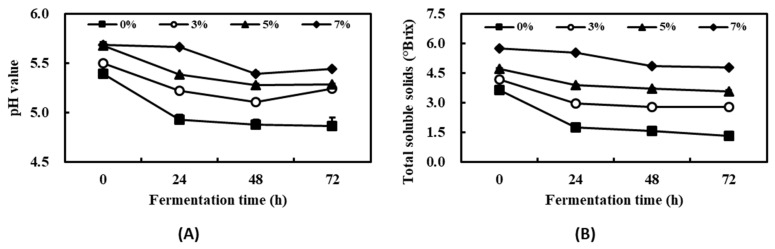
Changes in pH value (**A**) and total soluble solids (**B**) during the fermentation of kelp with *Saccharomyces cerevisiae*. Data are the mean ± S.D. of three independent measurements.

According to a report on the effect of seaweeds on agricultural products, many studies have shown that treatment with seaweeds (mostly extracts) increases crop production [28]. In this study, the main purpose of the metabolic processes of *S. cerevisiae* was not ethanol production, which would hinder plant growth [29]. Thus, kelp powder and defatted soybean extract were mixed and supplemented with molasses for use as a substrate. The changes in the physicochemical properties of kelp fermented with *S. cerevisiae* containing 0%, 3%, 5%, or 7% (*w*/*v*) kelp powder were evaluated every 24 h for 72 h (Figure 1A). During the fermentation time, the pH values at 0, 24, 48, and 72 h in the 3% group were 5.49, 5.22, 5.11, and 5.24, respectively. Figure 1B shows that the total soluble solids in the 3% group at 0, 24, 48, and 72 h were 4.15, 2.95, 2.78, and 2.79, respectively. All groups showed a slight reduction in pH and total soluble solids compared with the initial broth at 0 h. Several other studies on changes in physicochemical properties during fermentation with *S. cerevisiae* have observed similar reductions in pH values and total soluble solids [30,31]. The 3% kelp powder was chosen as the optimal condition for further studies, which considered industrial applications and production because the 5% and 7% fermented groups showed low medium fluidity induced by the moisture absorption of the kelp powder in the fermentor.

### 2.3. Changes to Amino Acid Contents in Fermented Kelp for Hydroponics

Limited nitrogen-source conditions induce a higher activation/metabolization of alcohol production than non-limiting nitrogen environments [32]. In addition, the exogenous application of a nitrogen source improves the secondary growth of stored roots in one-year-old *P. ginseng* [33]. For these reasons, the salt-reduced kelp powder and molasses were supplemented with defatted soybean extract as a natural nitrogen source. The composition of free amino acids in fermented kelp without defatted soybean extract comprised only aspartic acid (2.72 mg/100 g) and glutamic acid (5.33 mg/100 g), which suggests a lack of amino acids may have influenced the growth of the plant. Thus, to supply a nitrogen source in the fermented nutrient solution to enhance plant growth, the defatted soybean extract was added.

As shown in Table 2, the FK contained various free and protein-bound amino acids, including aspartic acid, glutamic acid, and alanine. The free amino acid contents in the FK showed significantly lower amounts of aspartic acid (6.59 mg/100 g), glutamic acid (12.59 mg/100 g), serine (0.41 mg/100 g), histidine (0.48 mg/100 g), glycine (0.45 mg/100 g), threonine (0.22 mg/100 g), arginine (0.79 mg/100 g), alanine (1.92 mg/100 g), tyrosine (0.15 mg/100 g), phenylalanine (0.16 mg/100 g), and lysine (0.76 mg/100 g) than the initial fermentation broth. This amounted to a 51.64% decrease in the total free amino acid contents relative to the initial broth. In contrast, the protein-bound amino acid contents in the FK showed significantly higher amounts of aspartic acid (59.19 mg/100 g), serine (15.66 mg/100 g), glycine (17.70 mg/100 g), threonine (15.40 mg/100 g), arginine (17.50 mg/100 g), alanine (20.91 mg/100 g), valine (17.13 mg/100 g), leucine (19.23 mg/100 g), and lysine (23.79 mg/100 g) than the initial fermentation broth. This was a 16.31% increase in the total protein-bound amino acid contents in the FK relative to the initial broth. Many studies have reported changes to the free and protein-bound amino acid compositions upon fermentation. Callejón et al. [34] reported that the contents of the free amino acids aspartic acid, glutamic acid, serine, glycine, threonine, arginine, and alanine were decreased in strawberry wine fermented with commercial yeast. In addition, Sarkar et al. [35] investigated changes in the free and protein-bound amino acids in kinema, a traditional Nepalese food made from fermented soybeans. In their results, the changes in amino acid compositions were shown by the reduction in free amino acids via metabolization and the elevation of protein-bound amino acids via the proteolytic activity of microorganisms. Therefore, this result suggests that the initial FK broth was effectively fermented by *S. cerevisiae*.

### 2.4. Total Phenolic Content and Total Flavonoid Content in the Extracts from Ginseng Sprouts Cultivated with Fermented Kelp

Ginseng contains various phenolic acids and flavonols such as gallic acid, chlorogenic acid, benzoic acid, epigallocatechin, catechin, and quercetin [1]. Table 3 shows the total phenolic content (TPC) and total flavonoid content (TFC) in the extracts of FK-treated GSs cultivated using a smart-farm system. The treatments with FK at the 0% (16.86 mg GAE/g), 10% (15.58 mg GAE/g), 25% (15.42 mg GAE/g), 50% (15.49 mg GAE/g), and 100% (15.16 mg GAE/g) concentrations showed significantly higher amounts of TPC in the GS extracts than the control group (11.62 mg GAE/g). Similarly, the TFC for the control group cultivated with tap water was 63.76 mg QE/g, and significant increases in TFC were seen for the 0% (70.31 mg QE/g) and 10% (69.71 mg QE/g) treatment concentrations, which were cultivated using 0.02% FK. The TPC and TFC in the extracts from the control group cultivated using tap water showed results similar to those in previous studies of GSs from plant factory cultivation systems [1,9,36]. In our study, however, the TPCs of the 0%, 10%, 25%, 50%, and 100% groups cultivated using 0.2% FK increased by 45.09%, 34.11%, 32.72%, 33.32%, and 30.44%, respectively, relative to the control group. This suggests that the treatment and supplementation with FK during cultivation affected the TPC and TFC in the GSs grown in this smart-farm system.

### 2.5. Ginsenosides in Extracts from Ginseng Sprouts Treated with Fermented Kelp

**Figure A1:**
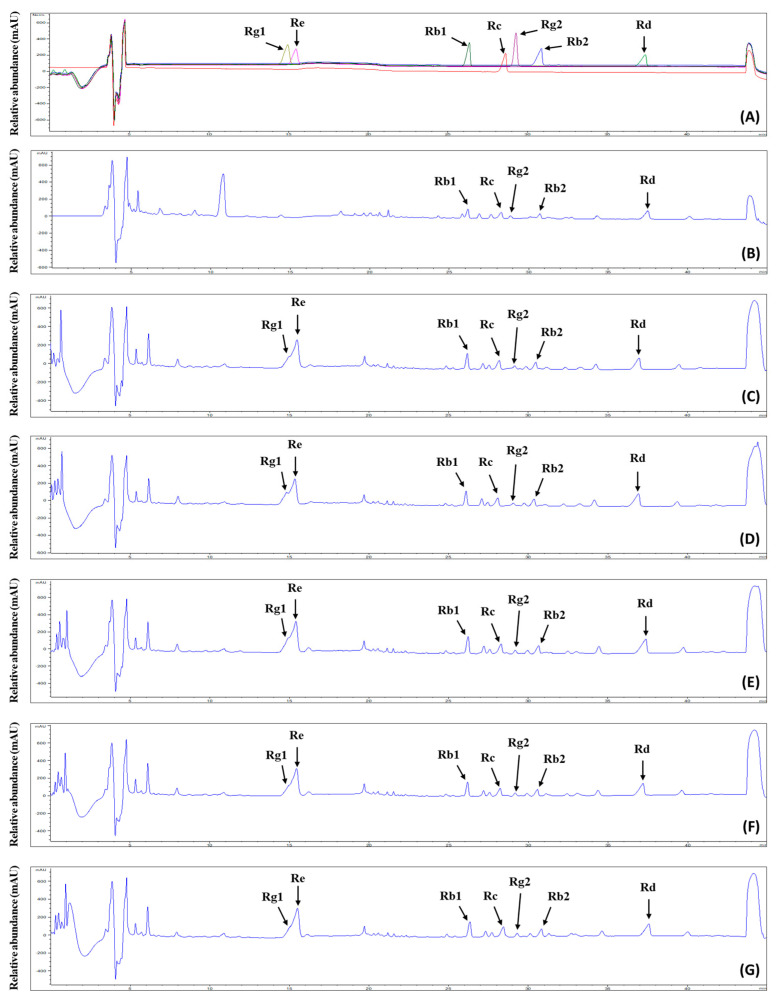
Chromatogram of ginsenosides as analyzed by HPLC for the ginsenoside-enriched extract fractions from ginseng sprouts treated with different concentrations of fermented kelp (FK). (**A**) Seven ginsenoside standards, different colors indicate chromatograms of seven ginsenosides, respectively; (**B**) control, cultivated using tap water; (**C**) 0% FK; (**D**) 10% FK; (**E**) 25% FK; (**F**) 50% FK; (**G**) 100% FK. Ginsenosides: 1, Rg1; 2, Re; 3, Rb1; 4, Rc; 5, Rg2; 6, Rb2; 7, Rd.

Ginsenosides are the representative bioactive constituents of *P. ginseng* species, in which over 120 kinds of ginsenosides have been identified, elucidating various biological activities with high added value [37]. For this reason, many processes such as the steaming and treatment of enzymes have been challenging for increasing the ginsenoside contents in ginseng. The analysis of our prepared GS extract fractions enriched with ginsenosides indicated that the control group contained 131.8 mg/g, while the 0%, 10%, 25%, 50%, and 100% groups contained 180.11 mg/g, 229.73 mg/g, 213.05 mg/g, 194.15 mg/g, and 192.72 mg/g, respectively. To identify seven ginsenosides (Rg1, Re, Rb1, Rc, Rg2, Rb2, and Rd), the ginsenoside-enriched fractions (GFs) were analyzed using a high-performance liquid chromatography-evaporative light scattering detector/mass spectrometry (HPLC-ELSD/MS) system. Figure A1 shows the ginsenoside chromatograms for the GFs from GS extracts cultivated using FK. Ginsenosides Rb1, Rc, Rg2, Rb2, and Rd were identified in the GFs of all groups. In addition, ginsenosides Rg1 and Re were detected in the FK-administered groups but not in the control group, which was cultivated using tap water. Various parameters, such as the linearity, limits of detection (LOD), and limits of quantification (LOQ), were investigated to determine the efficiency of the method. As shown in Table 4, a correlation coefficient (r^2^) value > 0.9968 indicated that the calibration curves for the seven ginsenosides exhibited high linearity. The LOD values ranged from 1.63 to 6.90, while the LOQ values ranged from 16.27 to 69.01 (Table 4). 

The seven ginsenoside contents in the experimental GFs are presented in Table 5. The Rg1 content ranged from 465.92 to 633.29 μg/g, the Re content ranged from 1499.75 to 1891.27 μg/g, the Rb1 content ranged from 330.46 to 518.23 μg/g, the Rc content ranged from 244.36 to 419.88 μg/g, the Rg2 content ranged from 54.28 to 66.94 μg/g, the Rb2 content ranged from 144.40 to 264.91 μg/g, and the Rd content ranged from 461.08 to 913.37 μg/g. The GSs cultivated using FK as a medium contained a higher amount of ginsenosides than the control group. In particular, the concentrations in the 25% group showed significantly higher amounts of the seven ginsenosides, with increases in Rb1, Rc, Rg2, Rb2, and Rd of 56.82%, 71.83%, 23.34%, 83.46%, and 98.09%, respectively, relative to the control group. Previous studies have reported on the effects of applying biostimulants and plant-growth-promoting rhizobacteria to plants. Mannino et al. [38] investigated the effects of a commercial biostimulant based on seaweed and yeast extracts on tomato growth and found that it improved harvest time and nutritional composition. Ashour et al. [39] reported that the treatment of hot pepper (*Capsicum annuum*) with a seaweed extract growth regulator induced significant increases in antioxidative substances such as ascorbic acid, TPC, and TFC. Similarly, Lam et al. [40] found increased bioactive constituents in *Agastache rugosa* treated with indole-3-acetic acid during hydroponic cultivation in a plant factory. Kim et al. [41] reported that a natural bioactive product composed of 50 kinds of plant materials elicited a significant elevation of ginsenoside accumulation in ginseng cultivated using an aeroponic system. Shukla et al. [42] investigated the effect of administering an extract from *Ascophyllum nodosum*, a brown seaweed, as a biostimulant in *Zea mays* under phosphorus-limited conditions. In the study, supplementation with the *A. nodosum* extract modulated the metabolic and signal transduction pathways with the expression of secondary metabolism genes. In the future, studies on gene expression relevant to secondary metabolism in GSs cultivated with FK will be required. However, our results indicate that the administration of FK effectively increases ginsenoside accumulation in GSs cultivated using smart farms.

## 3. Materials and Methods

### 3.1. Materials

Kelp was provided by Badapume Co., Ltd. (Wando, Republic of Korea). Molasses was purchased from Mosco International Commodities Pvt., Ltd. (Gujarat, India). One-year-old *P. ginseng* was obtained from the Elounsesang Agricultural Corp. (Yeonggwang, Republic of Korea).

### 3.2. Preparation of Kelp Powder

Raw kelp was thoroughly washed with high-pressured tap water to eliminate foreign substances, boiled for 1 min, and cooled to room temperature. It was then crushed using a screw-type crusher (Sungwon System Co., Ansan, Republic of Korea) and dried in a drying oven (Jeio Tech Co., Daejeon, Republic of Korea) for 20 h at 50 °C. The dried kelp was powderized with a grinder and stored at −20 °C until the experiments.

### 3.3. Salinity, pH, and Total Soluble Solids Determination

To evaluate the salinity of the raw and boiled kelp, 100 mL of distilled water (DW) was mixed and homogenized with 10 g of raw or boiled kelp, respectively. The homogenates were centrifuged, and the salinities of the supernatants were measured using a salt meter (Atago pocket PAL-SALT, Atago Co., Tokyo, Japan). The pH of the samples was analyzed using a pH meter (F-71, Horiba Co., Ltd., Kyoto, Japan), and the total soluble solid contents were measured using a saccharimeter (PAL-3, Atago Co., Japan).

### 3.4. Production of Fermented Kelp for Hydroponics

Fermentation using the kelp powder was performed three times by batch culture. To produce the FK for hydroponics, a 3% (*w*/*v*) solution of defatted soybean (Hokyoung-tech Co., Anseong, Korea) was extracted and filtered. The extract was then mixed with 2% (*w*/*v*) molasses and 0%, 3%, 5%, or 7% (*w*/*v*) kelp powder. These mixtures were pasteurized for 15 min at 121 °C. *Saccharomyces cerevisiae* (KCTC 17299, Korea Collection for Type Cultures, Jeongup, Korea) was inoculated into the mixtures at 0.01% (*v*/*v*), which were then incubated at 30 °C on a rotational shaker (200 rpm) for 72 h (KoBiotech Co., Ltd., Incheon, Korea). After the kelp fermentation, the fermentates were pasteurized for 30 min at 90 °C. The FK was then filtered using a 0.45 μm sterilized membrane filter and stored at 4 °C.

### 3.5. Cultivation of Ginseng Sprouts with Fermented Kelp in a Smart-Farm System

**Figure 2:**
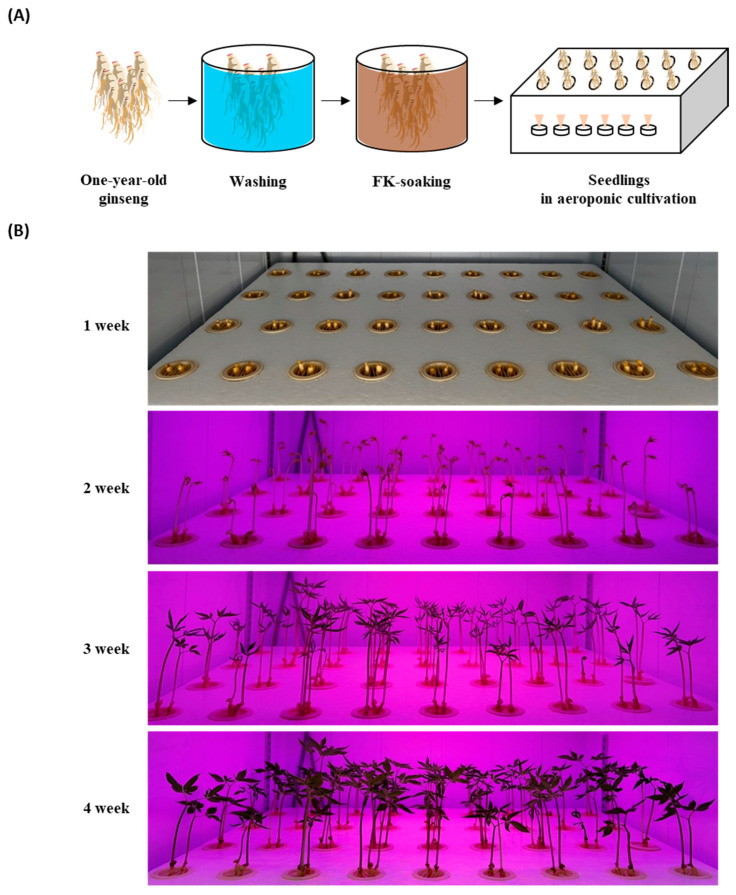
Cultivation of ginseng sprouts with fermented kelp (FK) using an aeroponic system. (**A**) Process of ginseng seedling and (**B**) growth status of ginseng sprouts in an aeroponic environment.

To compare the ginsenosides in smart-farm-cultivated GSs with FK, before planting, one-year-old ginsengs were soaked in one of five concentrations of FK (0%, 10%, 25%, 50%, or 100%) for 3 h (Table 6 and Figure 2A). The seedlings were planted in 530 × 405 × 35 mm (L × W × H) trays, and the medium was sprayed every 45 min for 1 min. The control group was planted without an FK soaking, and tap water was sprayed as the medium during the cultivation period (Table 6). All planted ginsengs were cultivated in the dark for 7 days at a temperature of 25 ± 2 °C and relative humidity of 70 ± 5%. They were then exposed to LED lamps for 21 days at a 142 μmol∙m^−2^·s^−1^ photosynthetic photon flux density and 2700 lx (lm/m^2^) illuminance as measured using a spectral PAR meter (PG200N, Zhunan, Taiwan). These experiments were conducted in a customized laboratory-scale smart-farm chamber (Mo Green Korea Co., Ltd., Gwangju, Korea). The cultivated GSs were harvested 28 days after plantation and stored at 4 °C until further experiments (Figure 2B).

### 3.6. Analysis of Free and Protein-Bound Amino Acids in Fermented Kelp

The sample preparation method used in this study was based on the method of Marino et al. [43] with some modifications. To determine the compositions of the protein-bound amino acids, 0.1 g of sample was added to a tube, and 1 mL of 0.05% (*v*/*v*) 2-mercaptoethanol (Sigma-Aldrich, St. Louis, MO, USA) in 6 N HCl was added. The tube was then vortex-mixed for 1 min, filled with nitrogen gas to remove the air, tightly capped, and placed in a drying oven (Jeio Tech Co.) at 95 °C for 24 h to hydrolyze the sample. After hydrolysis, the tubes were placed in the dark for 30 min to cool. The supernatant was then carefully transferred into a 50 mL volumetric flask, DW was added to reach 50 mL, and the mixture was filtered through a 0.2 µm syringe filter and transferred into a glass vial for analysis. To determine the free amino acid compositions, 1 g of sample and pure 0.02 N HCl were added to 50 mL volumetric flasks and sonicated for 30 min. The resulting mixtures were filtered through 0.2 µm syringe filters and transferred into glass vials for analysis.

The free and protein-bound amino acid composition analyses were conducted on an HPLC instrument (Agilent 1220, Agilent, Santa Clara, CA, USA). The analytical column was a Proshell HPH C18 (150 × 4.6 mm, 4 µm; Agilent); the analytical variable wavelength detector was set to measure a wavelength of 338 nm. The temperature of the column oven was 40 °C. The mobile phases A and B were 10 mM sodium phosphate dibasic and 10 mM sodium tetraborate decahydrate (1:1, *v*/*v*, pH 8.2) and acetonitrile, methanol, and water (4.5:4.5:1, *v*/*v*/*v*), respectively. Solvents A and B were run at a flow rate of 1.5 mL/min using a gradient of 98% A (2% B) at 0 min, steady at 98% A for 1.9 min, decreasing to 43% A over 16.2 min, decreasing to 20% A over 0.5 min, held steady at 20% A for 3.7 min, and then increasing to 98% A over 0.9 min. The column was equilibrated with 98% A for 3.7 min before the next injection. For the derivatization, O-phthalaldehyde (Sigma-Aldrich) and 3-mercaptopropionic acid (Sigma-Aldrich) in borate buffer (Agilent) were used.

### 3.7. Preparation of Ginseng Sprout Extracts

The six groups of GSs (control group treated with tap water and the 0%, 10%, 25%, 50%, and 100% FK-treated groups) were subjected to a heat reflux extraction with 70% (*v*/*v*) ethanol (800 mL) at 85 °C for 3 h and then filtered onto filter paper No. 2 (Advantec, Tokyo, Japan). The filtrates were concentrated up to dryness by a rotary vacuum evaporator (N-1200-B, Eyela, Tokyo, Japan), and the dried extracts were stored at −20 °C until further experiments.

### 3.8. Preparation of the Ginsenoside-Enriched Fraction

The GF was prepared using the methods of Shehzad et al. [44] with some modifications. Briefly, 1 g of GS extract was loaded into an open column (15 cm × 3 cm I.D.) packed with Diaion HP-20 (Sigma-Aldrich) and then sequentially eluted with an ethanol gradient beginning with 100% water and increasing to 20%, 70%, and finally 100% ethanol. The ginsenoside-rich 70% fractions were evaporated, lyophilized, and stored at −20 °C for the ginsenoside analysis.

### 3.9. Total Phenolic Contents

The TPC of the GS extracts was analyzed using the Folin–Ciocalteu colorimetric method [45] with some modifications. Briefly, the extracts were reacted with Folin–Ciocalteu reagent and neutralized with a sodium carbonate solution. The absorbance was then measured using a spectrophotometer at 760 nm (Epoch2, BioTek Co., Winooski, VT, USA). Gallic acid (Sigma-Aldrich, purity > 99%) was used as the standard, and the TPC is expressed as mg gallic acid equivalents/g (mg GAE/g).

### 3.10. Total Flavonoid Contents

The TFC of the GS extracts was evaluated with the aluminum chloride colorimetric method [46]. A 250 μL volume of sample was mixed with 100 μL of 1 M sodium nitrite and 1 mL of DW in a dark room for 5 min. Next, 150 μL of 5% aluminum chloride was added and reacted in the dark for 6 min. Volumes of 500 μL 10% sodium hydroxide and 500 μL DW were then added, and the solutions were incubated in the dark for a further 10 min. The absorbance of the reactants was measured at the 510 nm wavelength using a spectrophotometer (Epoch2, BioTek). Quercetin (Sigma-Aldrich, purity > 99%) was used as the standard, and the TFC is expressed as mg quercetin equivalents/g (mg QE/g).

### 3.11. Identification and Quantification of Ginsenosides in Ginseng Sprout Extracts

An HPLC-ELSD/MS analysis was performed to identify and quantify the ginsenosides. Mass spectra were analyzed at low resolution using an Agilent 6120 Quadrupole mass spectrometer coupled with an Agilent 1260 series HPLC, evaporative light scattering detector G4260A, and electrospray ionization source. The column used for the analysis was a Phenomenex C 18 (4.6 × 250 mm, 5 µm) column. The flow rate was maintained at 0.6 mL/min, and 10 µL samples prepared as in §3.8 were injected for the analyses. Solvents A (water) and B (acetonitrile) were used in the mobile phase under the following conditions: a hold period of 10 min with 23% solvent B as the initial solvent; a gradient condition increasing solvent B from 23% to 32% over 5 min; a gradient condition increasing solvent B from 32% to 80% over 28 min (min 15–43); and a final hold in 23% solvent B for 2 min. Solvents A and B were in 0.1% formic acid. The spectra analysis was performed at 203 nm.

Calibration curves were organized by plotting the peak areas using the concentrations of each ginsenoside. Standard solutions of seven ginsenosides (Rg1, R2, Rb1, Rc, Rg2, Rb2, and Rd; Sigma-Aldrich, purity > 99%) were prepared at 5 concentrations in the range of 62.5–1000 µg/mL. The seven standard ginsenosides were diluted with methanol to a series of appropriate concentrations and analyzed using the HPLC system. The limits of detection (LOD) and quantification (LOQ) were evaluated by linear regression with standard deviation (SD)/slope of the calibration curve (S) values of 3 and 10, respectively.

### 3.12. Statistical Analysis

Each experiment was conducted in triplicate, and all data are presented as the means ± standard deviation (S.D.). The analysis data were evaluated by a one-way analysis of variance using the software Static Analysis System ver. 9.4 (SAS Institute Inc., Cary, NC, USA) and by determining differences with the means using the Duncan multiple-range test (*p* < 0.05). 

## 4. Conclusions

In this study, a reduced-salt kelp powder was developed to produce an FK for hydroponics by fermenting kelp with *S. cerevisiae*. After the fermentation, the free and protein-bound amino acid compositions of the FK were evaluated by HPLC. The optimized FK was utilized in a hydroponic system as a biostimulant and medium. One-year-old ginseng administered FK and cultivated using a smart-farm system showed an elevation in TPC and TFC. Of particular note, the amounts of seven ginsenosides were significantly enhanced in the FK-administered groups, particularly the 25% concentration group. However, further studies on the molecular mechanisms in ginseng related to ginsenoside biosynthesis will be required to demonstrate the effects of FK during hydroponic cultivation. Overall, our results showing enhanced ginsenoside contents suggest that FK would be expected to be useful as a novel nutrient solution with high added values to cultivate ginsenoside-enriched GSs in hydroponics systems while reducing the use of chemical substances.

## Figures and Tables

**Table 1:** Changes in kelp salinity after a boiling process.

Contents	Raw Kelp	Boiled Kelp
Salinity (%)	1.97 ± 0.01	0.43 ± 0.01

Data are the mean ± S.D. of three independent measurements.

**Table 2:** Changes in free and protein-bound amino acid compositions in fermented kelp (FK).

Contents (mg/100 g)	Initial Broth	FK
Free amino acids		
Aspartic acid	11.45 ± 0.56 a	6.59 ± 0.02 b
Glutamic acid	28.17 ± 1.54 a	12.59 ± 0.06 b
Serine	0.58 ± 0.04 a	0.41 ± 0.05 b
Histidine	0.67 ± 0.03 a	0.48 ± 0.01 b
Glycine	0.52 ± 0.02 a	0.45 ± 0.02 b
Threonine	0.31 ± 0.02 a	0.22 ± 0.01 b
Arginine	2.30 ± 0.09 a	0.79 ± 0.02 b
Alanine	3.72 ± 0.18 a	1.92 ± 0.08 b
Tyrosine	0.40 ± 0.02 a	0.15 ± 0.01 b
Phenylalanine	0.54 ± 0.03 a	0.16 ± 0.01 b
Lysine	0.36 ± 0.01 b	0.76 ± 0.01 a
Total	50.77 ± 2.66	24.55 ± 0.31
Protein-bound amino acids		
Aspartic acid	55.34 ± 1.59 a	59.19 ± 2.61 a
Glutamic acid	76.62 ± 4.78 a	74.53 ± 3.38 a
Serine	11.22 ± 0.82 a	15.66 ± 2.72 a
Glycine	14.13 ± 0.38 b	17.70 ± 1.97 a
Threonine	11.25 ± 0.98 b	15.40 ± 0.98 a
Arginine	13.94 ± 0.64 b	17.50 ± 1.04 a
Alanine	17.21 ± 0.33 b	20.91 ± 0.86 a
Valine	11.91 ± 0.91 b	17.13 ± 2.22 a
Leucine	12.82 ± 0.82 b	19.23 ± 0.39 a
Lysine	17.56 ± 1.45 b	23.79 ± 0.67 a
Total	241.65 ± 12.43	281.04 ± 16.48

Data are the mean ± S.D. of three independent measurements. Different letters show the differences in Duncan multiple tests (*p* < 0.05) between values in the row.

**Table 3:** The phenolic and flavonoid contents in extracts from ginseng sprouts treated with fermented kelp (FK) and cultivated in a smart-farm system.

Contents	Control	Concentrations of FK Treatment				
		0%	10%	25%	50%	100%
TPC (mg GAE/g)	11.62 ± 0.25 c	16.86 ± 0.55 a	15.58 ± 0.21 b	15.42 ± 0.15 b	15.49 ± 0.41 b	15.16 ± 0.21 b
TFC (mg QE/g)	63.76 ± 1.36 b	70.31 ± 0.52 a	69.71 ± 1.55 a	58.71 ± 2.73 c	62.57 ± 2.36 b	63.17 ± 2.73 b

Data are the mean ± S.D. of three independent measurements. Different letters show the differences in Duncan multiple range tests (*p* < 0.05) between values in the row. GAE, gallic acid equivalent; QE, quercetin equivalent; TPC, total phenolic content; TFC, total flavonoid content.

**Table 4:** Calibration curves, limits of detection (LOD), and limits of quantification (LOQ) for seven ginsenosides.

Ginsenosides	Retention Time (min)	Calibration Curve	Correlation Coefficient (r^2^)	Test Range (mg/mL)	LOD (ng)	LOQ (ng)
Rg1	14.95	y = 6.3944x − 3.3708	0.9997	0.0125–1.000	1.63	16.27
Re	15.45	y = 4.399x − 168.34	0.9991	0.0125–1.000	5.62	56.23
Rb1	26.36	y = 4.2953x − 8.8375	0.9995	0.0125–1.000	6.90	69.01
Rc	28.62	y = 4.2794x + 63.467	0.9992	0.0125–1.000	3.27	32.67
Rg2	29.27	y = 6.7113x + 45.192	1.0000	0.0125–1.000	2.11	21.08
Rb2	30.86	y = 4.9103x + 47.146	0.9998	0.0125–1.000	3.97	39.74
Rd	37.38	y = 4.3557x + 21.278	0.9998	0.0125–1.000	4.34	43.38

Calibration curve: y equals peak area, and x equals concentration (mg/mL). Standard deviation (SD)/slope of the calibration curve (s) = 3; SD/s = 10.

**Table 5:** Ginsenoside contents in the ginsenoside-enriched extract fractions from ginseng sprouts treated with different concentrations of fermented kelp (FK).

Ginsenosides (μg/g)	Control	FK-Treatment Concentrations				
		0%	10%	25%	50%	100%
Rg1	N.D.	494.62 ± 2.72 b	465.92 ± 30.23 b	633.29 ± 33.05 a	501.27 ± 77.82 b	499.92 ± 112.21 b
Re	N.D.	1514.57 ± 53.92 b	1607.49 ± 43.14 b	1891.27 ± 1.11 a	1499.75 ± 78.87 b	1520.45 ± 140.77 b
Rb1	330.46 ± 71.94 c	427.81 ± 0.44 b	418.82 ± 7.27 b	518.23 ± 14.38 a	400.43 ± 0.83 b	503.58 ± 18.10 a
Rc	244.36 ± 2.84 d	359.61 ± 79.64 ab	324.59 ± 44.01 bc	419.88 ± 31.98 a	292.23 ± 11.12 bd	387.26 ± 70.88 ab
Rg2	54.28 ± 1.59 b	63.29 ± 0.89 a	56.29 ± 9.09 b	66.94 ± 2.28 a	62.60 ± 1.78 a	65.60 ± 7.37 a
Rb2	144.40 ± 3.13 e	197.98 ± 3.91 d	206.64 ± 0.51 c	264.91 ± 2.41 a	195.43 ± 2.83 d	239.43 ± 7.37 b
Rd	461.08 ± 2.11 e	599.19 ± 1.31 d	757.27 ± 11.07 b	913.37 ± 22.32 a	681.76 ± 3.05 c	687.55 ± 10.11 c

Data are the mean ± S.D. of three independent measurements. Different letters show the differences in Duncan multiple range tests (*p* < 0.05) between values in the row. N.D., not detected.

**Table 6:** Fermented kelp treatments of ginseng sprouts cultivated using a smart-farm system.

Factors	Control	Concentrations of FK				
		0%	10%	25%	50%	100%
FK soaking	−	−	+	+	+	+
Aeroponic medium	Tap water	0.2% FK	0.2% FK	0.2% FK	0.2% FK	0.2% FK

## Data Availability

Data are contained within the article.
